# G Protein-Coupled Receptors in Cerebrovascular Diseases: Signaling Mechanisms and Therapeutic Opportunities

**DOI:** 10.3390/ijms27020736

**Published:** 2026-01-11

**Authors:** Qiuxiang Gu, Jia Yao, Jiajing Sheng, Dong Liu

**Affiliations:** Nantong Laboratory of Development and Diseases, School of Life Sciences, Co-Innovation Center of Neuroregeneration, Nantong University, Nantong 226019, China; qiuxiang@ntu.edu.cn (Q.G.); yj815258027@126.com (J.Y.)

**Keywords:** GPCR, NVU, cerebrovascular diseases, targeted therapy

## Abstract

G protein-coupled receptors (GPCRs) are key regulators of cerebrovascular function, integrating vascular, inflammatory, and neuronal signaling within the neurovascular unit (NVU). Increasing evidence suggests that GPCR actions are highly dependent on cell type, signaling pathway, and disease stage, leading to distinct, and sometimes opposing, effects during acute ischemic injury and post-stroke recovery. In this review, we reorganize GPCR signaling mechanisms using a disease-stage-oriented and NVU-centered framework. We synthesize how GPCR-mediated intercellular communication among neurons, glial cells, and vascular elements dynamically regulates cerebral blood flow, neuroinflammation, blood–brain barrier (BBB) integrity, and neuronal circuit remodeling. Particular emphasis is placed on phase-dependent GPCR signaling, highlighting receptors whose functions shift across acute injury, secondary damage, and recovery phases. We further critically evaluated the translational implications of GPCR-targeted therapies, discussing why promising preclinical neuroprotection has frequently failed to translate into clinical benefit. By integrating molecular mechanisms with temporal dynamics and translational constraints, this review provides a framework for the rational development of cell-type and stage-specific GPCR-based therapeutic strategies in cerebrovascular disease.

## 1. Introduction

Cerebrovascular diseases remain a leading cause of mortality and long-term disability worldwide, with ischemic stroke representing the most prevalent and devastating manifestation [1,2,3]. Despite substantial advances in acute reperfusion therapies, effective strategies to limit secondary injury and promote long-term functional recovery remain limited [4,5,6,7]. A deeper understanding of the molecular signaling pathways governing cerebrovascular dysfunction is therefore essential.

G protein-coupled receptors (GPCRs) constitute the largest family of signal transduction receptors and regulate diverse physiological processes relevant to cerebrovascular pathology, including vascular tone, immune activation, and neuronal survival [8,9,10]. However, GPCR signaling in the brain is inherently complex. The same receptor may engage distinct downstream pathways depending on cell type, ligand availability, and microenvironmental conditions, leading to context-dependent and sometimes contradictory biological outcomes [11,12,13,14]. This complexity has contributed to inconsistent interpretations across studies and has hindered successful clinical translation.

Most existing reviews describe GPCRs primarily in a receptor-centered or single-cell-type manner, often emphasizing vascular smooth muscle cells (VSMCs), endothelial cells, or the blood–brain barrier (BBB) in isolation [15]. Such approaches insufficiently capture the coordinated, multicellular signaling processes that underlie cerebrovascular injury and recovery. Increasing evidence indicates that neurons, glial cells, vascular cells, and infiltrating immune cells function as an integrated neurovascular unit (NVU), in which intercellular communication critically shapes both acute injury and repair responses [16]. Moreover, the temporal dynamics of GPCR signaling across acute ischemia, secondary injury, and post-stroke repair phases are rarely addressed in an integrated manner.

In this review, we adopt a disease-process-oriented framework to systematically examine GPCR signaling in cerebrovascular disorders. By incorporating an NVU-centered perspective and emphasizing temporal phase-specific effects, we aim to clarify how GPCR signaling contributes to injury progression and recovery, and to identify conceptual principles relevant for therapeutic development.

## 2. Canonical GPCR Signaling Pathways

### 2.1. GPCRs and the G Protein Family

GPCRs, a large superfamily conserved from yeast to humans, mediate most signaling pathways and sensory functions, with ~800 mammalian genes and ~30% of FDA-approved drugs [17]. Despite the structural diversity of GPCR ligands (from photons to peptides), all ligands induce GPCR conformational changes to activate G proteins. Structurally, all GPCRs share an extracellular N-terminus, seven transmembrane α-helices, three extracellular loops, three intracellular loops, and a cytoplasmic C-terminus [18]. Phylogenetic analysis classifies human GPCRs into five major families with distinct functions:

- Rhodopsin family (Class A): The largest subgroup (~70% of GPCRs), including adrenergic receptors, endothelin receptors, and chemokine receptors (e.g., CCR5, CXCR4).
- Secretin family (Class B): Ligands are peptide hormones (e.g., glucagon), with receptors involved in metabolic regulation.
- Glutamate family (Class C): Includes metabotropic glutamate receptors (mGluRs) and γ-aminobutyric acid (GABA) receptors, critical for neural signaling.
- Adhesion family: Regulates cell adhesion and tissue development, with roles in vascular integrity.
- Frizzled/Taste2 family: Mediates Wnt signaling (Frizzled) and taste perception (Taste2).

G proteins are subdivided into Gαs, Gαi/o, Gαq/11, and Gα12/13 families. Ligand binding to GPCRs induces GTP loading on Gα and dissociation from Gβγ, enabling both subunits to engage downstream effectors.

### 2.2. Core GPCR Signal Transduction Mechanisms

**Figure 1:**
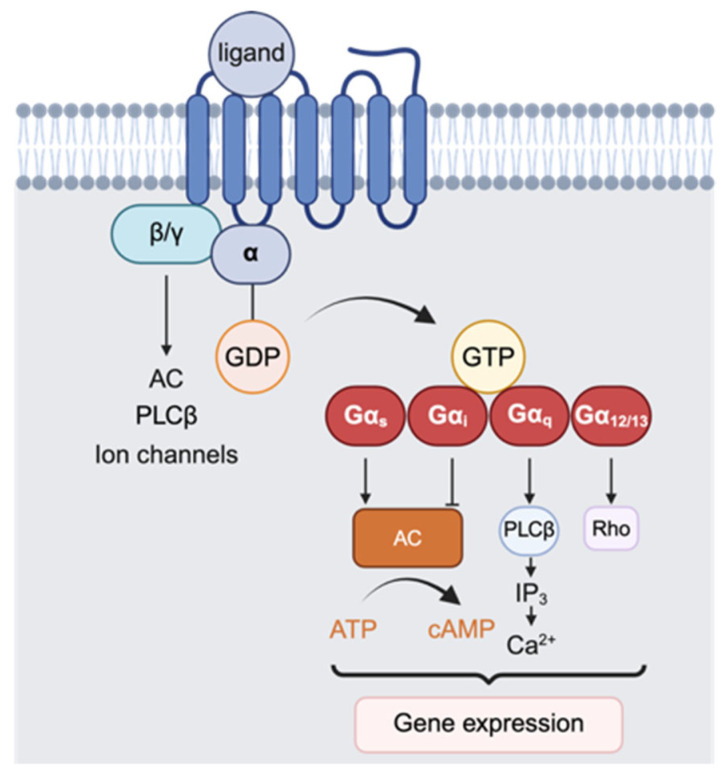
GPCR-G protein complexes in signal transduction. GPCRs activate heterotrimeric G proteins via GDP-GTP exchange on Gα, releasing Gα and βγ to regulate effectors like AC, PLCβ, and Rho, modulating cAMP, Ca^2+^, and gene expression. This figure was created with BioRender (agreement number: OT293PX79F).

The main effectors of G proteins include the adenylate cyclase (AC) system, phospholipase C (PLC), and others. Different G protein families activate their respective effectors, thereby initiating downstream signaling pathways (Figure 1).

Gαs activates AC to convert ATP into cAMP, which stimulates PKA and cyclic nucleotide-gated channels, whereas Gαi inhibits AC, reducing cAMP levels [19]. Gαq activates phospholipase Cβ (PLCβ) to hydrolyze PIP_2_ into IP_3_ and DAG; IP_3_ triggers Ca^2+^ release from the endoplasmic reticulum, and DAG activates PKC, inducing downstream cellular responses [20]. Members of the RGS (Regulator of G-protein Signaling) family can activate the GTPase activity of specific Gα subunits. p115RhoGEF, a guanine nucleotide exchange factor for Rho proteins, can specifically stimulate the GTPase activity of Gα12/13 in G proteins [21]. Additionally, Gα12 can exert its functions by interacting with receptor tyrosine kinase family members, cadherins, and other proteins [22]. In addition to Gα proteins, Gβγ proteins can also regulate AC [23,24], PLCβ [25,26], inward rectifier K^+^ channels [27,28], and voltage-gated Ca^2+^ channels [29,30], thereby facilitating signal transduction.

These signaling pathways regulate intracellular cAMP production, Ca^2+^ release, PKC activation, and RhoA-dependent cytoskeletal remodeling—mechanisms highly relevant to vascular tone, endothelial permeability, and inflammatory activation.

## 3. GPCR Signaling Within the NVU

Within the NVU, GPCR signaling is organized across distinct cell types to enable coordinated responses to neuronal activity, metabolic stress, and inflammation [31]. Rather than functioning as isolated components, GPCRs form intercellular signaling axes that couple vascular, immune, and neuronal functions [31]. The functional outcome of GPCR activation depends on both cellular context and disease stage, so that disruption of these signaling networks contributes to neurovascular dysfunction and impaired recovery [32,33].

### 3.1. Endothelial–Pericyte GPCR Crosstalk in Vascular Stability

Endothelial cells and pericytes form the structural and functional backbone of the cerebral microvasculature. GPCR signaling between these two cell types regulates capillary tone, vascular permeability, and microvascular stability [34,35]. Endothelial GPCRs respond to circulating ligands such as adenosine [36], sphingosine-1-phosphate (S1P) [37,38], and prostaglandins [39], thereby modulating nitric oxide production, cytoskeletal dynamics, and junctional integrity. These endothelial responses, in turn, influence pericyte contractility and capillary perfusion.

Conversely, pericyte-expressed GPCRs sense local metabolic and neurotransmitter cues and relay signals back to endothelial cells to fine-tune microvascular resistance [40]. Disruption of this bidirectional GPCR-mediated communication destabilizes capillary networks and predisposes the vasculature to hypoperfusion and leakage during cerebrovascular injury.

### 3.2. Astrocyte–Endothelial GPCR Signaling at the BBB

Astrocytes play a pivotal role in regulating BBB function through close physical and biochemical interactions with endothelial cells. Astrocyte-derived ligands, including S1P [37,38], prostaglandins [39], and neurotransmitter metabolites [40], activate endothelial GPCRs to modulate tight junction organization and transcellular transport pathways. Through these mechanisms, astrocytic GPCR signaling indirectly governs BBB permeability and endothelial responsiveness to injury [32].

Endothelial GPCR activation also feeds back to astrocytes by altering extracellular ion balance and metabolic substrate availability, thereby influencing astrocytic reactivity [31]. This reciprocal GPCR-mediated signaling axis establishes a regulatory loop that maintains BBB homeostasis under physiological conditions and determines barrier vulnerability under pathological stress.

### 3.3. Microglia–Neuron GPCR Communication in Immune Surveillance

Microglia serve as the resident immune cells of the central nervous system and continuously monitor neuronal activity and tissue integrity. GPCRs expressed on microglia detect extracellular nucleotides, chemokines, and neurotransmitters released by stressed or active neurons [41]. Activation of these receptors shapes microglial surveillance behavior, cytokine secretion, and phagocytic activity [42].

Neuronal GPCR signaling reciprocally responds to microglia-derived mediators, thereby modulating neuronal excitability and stress responses [43]. This bidirectional GPCR-dependent communication ensures adaptive immune surveillance under homeostatic conditions but may become maladaptive when dysregulated, contributing to inflammatory amplification in cerebrovascular disease [44].

### 3.4. Integrated GPCR Networks Across the NVU

Although individual GPCR pathways can be assigned to specific NVU cell types, their functional consequences emerge from integrated network behavior. Endothelial activation alters immune cell recruitment [45], microglial responses influence vascular permeability [32], and astrocytic signaling shapes both vascular and neuronal function [46,47]. GPCRs act as nodal regulators within this network, enabling rapid and coordinated adaptation to metabolic and inflammatory cues.

**Figure 2:**
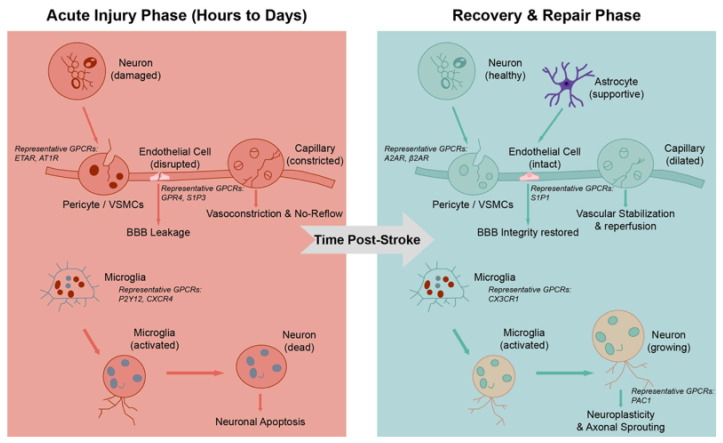
Spatiotemporal shifts in GPCR signaling within the NVU during stroke progression. Acute injury phase (hours to days): deleterious GPCR signaling predominates. Neuronal signals activate pericyte/VSMC GPCRs (e.g., ETAR, AT1R), leading to vasoconstriction and no-reflow [51,52,53,54,55]. Concurrently, endothelial GPCRs (e.g., GPR4, S1P3) drive BBB leakage, while microglial GPCRs (e.g., P2Y12, CXCR4) promote neuroinflammation and neuronal apoptosis (red arrows) [45,56,57,58,59]. Recovery phase (Repair): GPCR signaling shifts toward protective pathways. Neuronal activity engages pericyte/VSMC GPCRs (e.g., A2AR, β2AR) to promote vascular stabilization [60,61,62,63,64,65,66,67]. Astrocyte–endothelial crosstalk via GPCRs (e.g., S1P1) restores BBB integrity [37,38,68,69]. Microglial (e.g., CX3CR1) and neuronal (e.g., PAC1, A2AR) GPCR signaling facilitates inflammation resolution, synaptic plasticity, and axonal spouting (green arrows) [60,61,62,63,64,65,70,71]. Abbreviations: AT1R, angiotensin II type 1 receptor; S1P3, sphingosine-1-phosphate receptor 3; A2AR, adenosine A2A receptor; β2AR, β2-adrenergic receptor.

The repeated involvement of GPCRs such as S1P, PGE2, and A2AR across endothelial, glial, and neuronal compartments does not reflect redundancy, but rather context-specific signal partitioning within the NVU (Figure 2) [36,37,38,48,49,50]. By coupling to distinct intracellular effectors in different cell types, these receptors enable parallel regulation of vascular stability, immune activation, and neuronal excitability. This multi-level integration provides a mechanistic explanation for the pronounced context- and stage-dependence of GPCR signaling observed in cerebrovascular disease.

This NVU-centered view provides a conceptual framework for understanding how GPCR signaling synchronizes vascular, immune, and neuronal processes. It also highlights why therapeutic modulation of GPCRs often yields context-dependent outcomes, as perturbation of one signaling node can propagate across multiple NVU components.

## 4. GPCRs in Acute Cerebrovascular Injury

Acute cerebrovascular injury, particularly ischemic stroke, triggers a rapid cascade of pathological events, including cerebral hypoperfusion, sterile inflammation, and BBB disruption. GPCR signaling plays a central role in coordinating these processes through tightly regulated yet highly context-dependent actions across NVU cell types. Importantly, during the acute phase, GPCR-mediated responses frequently exert dual effects, with protective or deleterious outcomes determined by receptor subtype, cellular localization, and temporal window of activation [32,33].

### 4.1. Regulation of Cerebral Blood Flow During Acute Ischemia

Cerebral blood flow dysregulation is a defining feature of acute ischemic stroke [72]. GPCRs expressed on VSMCs and pericytes critically regulate vessel tone and microvascular perfusion during this phase. Activation of Gs-coupled GPCRs, including A2AR and prostaglandin E2 receptors EP2 and EP4, promotes cAMP-dependent vasodilation, thereby facilitating collateral circulation and partial restoration of perfusion in ischemic regions [50,66]. Experimental evidence further indicates that EP2/EP4 signaling supports functional hyperemia and capillary recruitment, consistent with a protective role in acute ischemia [73].

In contrast, activation of Gq- and G12/13-coupled GPCRs, such as AT1R, endothelin receptors, and prostaglandin F2α receptor (FP), induces intracellular Ca^2+^ elevation and RhoA-mediated contractile signaling [54,74,75]. These pathways exacerbate vasoconstriction, impair microvascular reperfusion, and contribute to the no-reflow phenomenon observed after recanalization. The balance between these opposing GPCR signaling axes critically shapes perfusion heterogeneity during the hyperacute stage of stroke and influences infarct expansion.

### 4.2. GPCR-Mediated Neuroinflammation and Secondary Injury

Neuroinflammation rapidly emerges following ischemic insult and represents a major contributor to secondary brain injury [76]. GPCRs expressed on endothelial cells, microglia, and infiltrating immune cells coordinate leukocyte recruitment, cytokine production, and inflammatory amplification within the ischemic microenvironment.

Endothelial GPCR activation regulates the initial inflammatory interface between circulating immune cells and the cerebral vasculature. For example, the proton-sensing GPCR GPR4 responds to ischemia-induced acidosis by enhancing endothelial activation, leukocyte adhesion, and vascular permeability, thereby facilitating immune cell infiltration [45]. Concurrently, microglial GPCRs, including chemokine and purinergic receptors, modulate inflammatory polarization and cytokine release, indirectly influencing neuronal vulnerability [77].

These GPCR-driven inflammatory responses do not occur in isolation but form feed-forward signaling loops across NVU components. Endothelial activation promotes immune cell entry, which in turn amplifies microglial reactivity and further disrupts vascular homeostasis. During the acute phase, excessive or prolonged activation of pro-inflammatory GPCR signaling exacerbates tissue damage, underscoring the importance of temporally controlled modulation rather than indiscriminate suppression of inflammation [77,78].

### 4.3. GPCR Regulation of BBB Disruption in the Acute Phase

BBB breakdown is a hallmark of acute cerebrovascular injury and a major contributor to cerebral edema and hemorrhagic transformation [79]. GPCR signaling influences BBB integrity primarily through rapid modulation of endothelial cytoskeletal dynamics and tight junction organization during the early injury phase [80,81,82].

Activation of GPCRs such as S1P3, protease-activated receptors (PARs), and bradykinin B2 receptors (B2Rs) promotes RhoA-ROCK signaling, actin stress fiber formation, and junctional disassembly, thereby increasing paracellular permeability [58,83]. These pathways exacerbate vascular leakage under ischemic and inflammatory conditions. In contrast, endothelial S1P1 signaling counteracts barrier disruption by stabilizing tight junctions and limiting cytoskeletal contraction, underscoring receptor subtype-specific effects within the same ligand system [37].

Notably, acute BBB disruption is mechanistically distinct from later-stage barrier remodeling and repair. During the acute phase, GPCR-mediated signaling primarily determines the severity and duration of barrier breakdown, thereby shaping early edema formation and secondary injury progression.

## 5. GPCRs in Neuronal Survival, Plasticity, and Functional Recovery

Beyond their established roles in acute vascular regulation, GPCRs critically shape long-term outcomes by orchestrating neurovascular remodeling. During the subacute and recovery phases, GPCR signaling transitions from injury mitigation to the promotion of neuronal resilience, synaptic plasticity, and circuit reorganization [84]. Importantly, these processes are not driven solely by neurons but rely on coordinated signaling among neurons, astrocytes, and remodeling vessels.

### 5.1. GPCR Regulation of Delayed Neuronal Survival

Following the acute insult, neurons in the ischemic penumbra remain vulnerable to delayed apoptosis and metabolic stress. During this window, GPCRs function as molecular “survival switches” that modulate cell fate decisions.

GPR37, a Gi-coupled receptor enriched in neurons and oligodendrocytes, acts as an endogenous neuroprotective brake [85]. Genetic deletion of GPR37 exacerbates ischemic injury by promoting caspase-3 activation and impairing mTOR signaling [85].

Furthermore, proton-sensing GPCRs link the acidic ischemic microenvironment to adaptive survival responses. TDAG8 (GPR65), although predominantly expressed in immune and glial cells, exerts paracrine neuroprotective effects, whereas neuronal GPR68 senses acidosis to trigger PKC-dependent survival pathways [86,87]. These observations highlight how GPCRs decode metabolic cues, such as extracellular pH, to preserve neuronal integrity during the subacute phase.

### 5.2. GPCR Modulation of Synaptic Plasticity and NVU Remodeling

Functional recovery after stroke depends on synaptic reorganization, a process tightly regulated by interactions between neurons and astrocytes within the tripartite synapse. GPCRs modulate this form of plasticity by controlling dendritic excitability, neurotransmission, and neurotrophic support.

The chemokine receptor CCR5 acts as a critical molecular “brake” on post-stroke plasticity [88,89]. Inhibition of CCR5 enhances motor recovery by disinhibiting CREB-dependent transcriptional programs that drive axonal remodeling and synaptic strengthening [88,89]. This finding represents a paradigm shift, suggesting that suppressing maladaptive GPCR signaling can be as crucial as activating pro-repair pathways during recovery.

Additionally, neuropeptide signaling via PACAP/PAC1 exemplifies coordinated neuro-glial coupling within the NVU [71]. PAC1 activation not only enhances neuronal excitability via Kv4.2 channels but also modulates astrocytic support for synaptic strengthening [90]. Such coordinated signaling ensures that circuit rewiring is accompanied by appropriate metabolic and trophic support from surrounding NVU components.

### 5.3. Stage-Dependent GPCR Signaling Switches and Functional Outcomes

A recurring challenge in translational research is the pronounced context dependence of GPCR signaling. The same receptor may exert opposing effects depending on disease stage, cell type, and duration of activation. β2ARs exemplify this duality. While β2AR activation may alter acute inflammation by inhibiting NF-κB, prolonged activation during the recovery phase may alter neuronal excitability and compromise network stability [91,92].

Similarly, GPCRs that drive pro-inflammatory responses during the acute phase may become necessary at later stages to facilitate debris clearance, synaptic remodeling, and remyelination [93]. These observations emphasize that GPCRs function as dynamic modulators rather than static therapeutic targets. Effective intervention, therefore, requires precise targeting of defined temporal windows, rather than blanket activation or inhibition.

Collectively, failure to account for these temporal switches can adversely affect functional outcomes. GPCR interventions that are beneficial for acute neuroprotection may impair cognitive recovery or long-term network stability if applied during later phases, highlighting the need for temporally optimized strategies to maximize sustained motor and cognitive rehabilitation.

## 6. Translational and Therapeutic Challenges of Targeting GPCRs in Cerebrovascular Diseases

**Figure 3:**
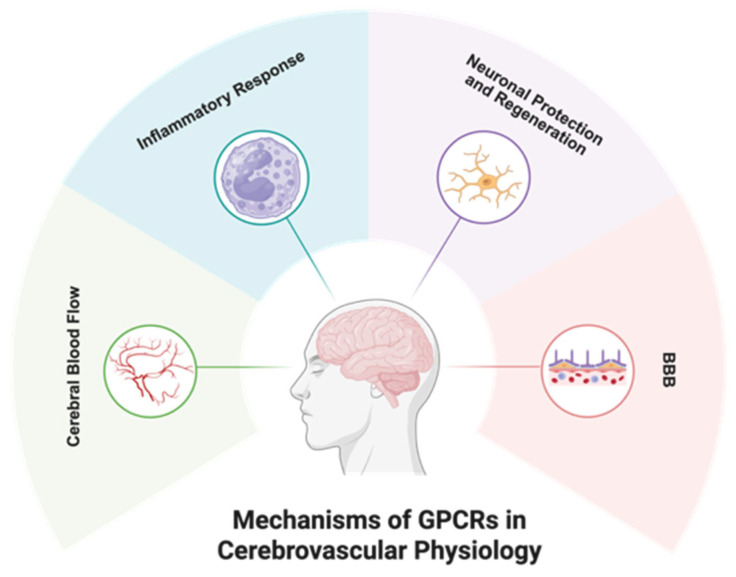
GPCR-mediated regulatory mechanisms in cerebrovascular pathologies. In stroke pathogenesis, GPCRs play central roles in cerebrovascular perfusion, neuroimmune responses, BBB homeostasis, and neuronal survival mechanisms. This figure was created with BioRender (agreement number: BU293PX64P).

GPCRs regulate multiple pathological processes in cerebrovascular diseases, including dysregulation of cerebral blood flow, neuroinflammation, BBB breakdown, and neuronal injury (Figure 3). Accordingly, GPCRs have emerged as attractive therapeutic targets in experimental stroke models. However, despite robust neuroprotective effects reported in rodents, the clinical translation of GPCR-targeted strategies has largely been unsuccessful.

### 6.1. Clinically Validated and Repurposed GPCR Targets

Several GPCR families have been investigated as therapeutic targets in stroke (Table 1).

❿ Adenosine receptors (ARs)

ARs regulate cerebral perfusion, inflammation, neuronal excitability, and BBB stability. Adenosine A1 receptor (A1AR) activation reduces excitotoxicity, while A2AR modulation promotes vasodilation and can limit ischemic injury in preclinical models [118,123]. Selective A2AR antagonists, such as istradefylline, approved for PD, are being explored for post-stroke neuroprotection due to their anti-inflammatory and neuroprotective potential [124,125].

❿ Endothelin receptors (ETRs)

ETARs mediate ET-1-induced cerebral vasoconstriction and contribute to ischemic damage [126]. Bosentan, a dual endothelin receptor antagonist, improves cerebral blood flow in preclinical ischemia models but may exacerbate systemic or infectious complications, highlighting the need for context-specific targeting strategies [52,127].

❿ Sphingosine-1-phosphate receptors (S1P receptors)

S1P signaling influences BBB integrity, neuroinflammation, and neurogenesis. FTY720 (fingolimod) activates S1P receptors on neurons, glia, and endothelial cells, promoting anti-apoptotic pathways and facilitating the proliferation of neural precursor cells [128,129]. In this regard, S1P-targeted therapies exemplify the multicellular actions of GPCRs within the NVU.

❿ Chemokine receptors (CCR5 and CXCR4)

These GPCRs regulate immune cell recruitment and post-stroke neuroplasticity [130]. CCR5 antagonism with maraviroc enhances motor and cognitive recovery in rodent models, and emerging human data support its potential for post-stroke neural repair [88,89,131]. In parallel, its therapeutic applications in long COVID/post-acute sequelae of COVID-19 are being evaluated in ongoing or planned clinical trials [132].

❿ Serotonin receptors (5-HTRs)

Several serotonin receptor subtypes modulate cerebrovascular tone. Activation of 5-HT1 and 5-HT2 receptors reduces cerebrovascular constriction, while 5-HT3 functions as a ligand-gated ion channel [119,120]. Agents such as sumatriptan, widely used in migraine therapy, influence cerebrovascular dynamics and may hold relevance for cerebrovascular modulation [121,122].

### 6.2. Discrepancy Between Preclinical Efficacy and Clinical Outcomes

A recurring challenge in GPCR-targeted stroke therapy is the poor concordance between preclinical neuroprotection and clinical efficacy [133]. Broad-spectrum modulation of adenosine receptors exemplifies this translational gap. In experimental ischemia, pharmacological antagonism or genetic deletion of A2ARs reduces infarct volume, attenuates neuroinflammation, and preserves synaptic integrity [65,134,135]. However, clinical attempts to modulate adenosine signaling have been constrained by narrow therapeutic windows, cardiovascular side effects, and context-dependent disruption of BBB integrity [136,137].

Similar discrepancies have been observed for other GPCR systems, including endothelin and chemokine receptors, which demonstrate protective effects in tightly controlled experimental paradigms but yield inconsistent or adverse outcomes in heterogeneous patient populations [138]. These findings indicate that GPCR-mediated neuroprotection is highly dependent on injury stage, cell type, and signaling context, and cannot be reliably extrapolated from simplified models lacking temporal and spatial resolution.

### 6.3. Pharmacological Barriers: BBB Penetration, Off-Target Effects, and Therapeutic Windows

Pharmacokinetic and pharmacodynamic limitations further impede the clinical translation of GPCR-targeted therapies [139]. Many GPCR ligands fail to achieve effective concentrations within ischemic brain tissue due to restricted BBB permeability or active efflux by transporters such as P-glycoprotein [140]. Even when BBB penetration is achieved, systemic administration frequently leads to off-target effects, particularly within the peripheral vasculature, resulting in hypotension or impaired cerebral autoregulation [141].

Moreover, the temporal dynamics of GPCR signaling present a fundamental challenge. GPCRs such as A2ARs and β2ARs exhibit phase-dependent effects, conferring vascular or anti-inflammatory benefits during early ischemia while potentially exacerbating BBB leakage or immune activation at later stages [65,91,92]. Therapeutic interventions that fail to account for these shifting windows may inadvertently convert protective signaling into deleterious outcomes [142,143].

### 6.4. Species Differences and Limitations of Rodent Models

Species-specific differences in GPCR expression patterns, coupling preferences, and signaling bias further limit translational predictability. GPCRs implicated in cerebrovascular pathology, including A2ARs, CXCR4, and β2ARs, exhibit divergent cell-type distributions and downstream signaling cascades in rodents compared with humans, particularly within endothelial cells, microglia, and pericytes [65,91,92,106]. These discrepancies likely contribute to the frequent failure of GPCR-targeted neuroprotective strategies during clinical translation.

In addition, commonly used rodent stroke models inadequately capture the vascular comorbidities, aging, and immune heterogeneity characteristic of human cerebrovascular diseases [144]. Consequently, GPCR-dependent mechanisms identified in young, otherwise healthy animals may not accurately reflect receptor function in the diseased human brain.

### 6.5. Emerging Human-Relevant Models for GPCR Research

To bridge this translational gap, human-relevant experimental platforms are increasingly being adopted. Induced pluripotent stem cell (iPSC)-derived NVU models enable controlled investigation of GPCR signaling across human neurons, astrocytes, endothelial cells, and pericytes within an integrated multicellular context [145,146]. Brain organoids and microfluidic NVU-on-chip systems further permit spatiotemporal modeling of ischemia–reperfusion injury and intercellular GPCR-mediated communication under human-specific conditions [147].

**Figure 4:**
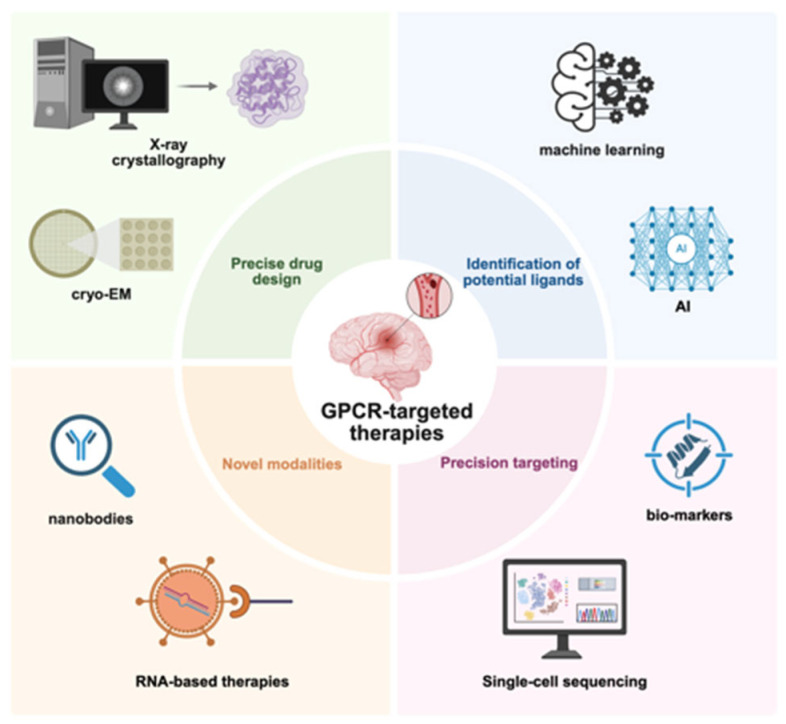
Discovery of GPCR-targeted agents against cerebrovascular pathologies. GPCR-targeted drug discovery advances through cryo-EM/X-ray structural studies, high-throughput AI-assisted ligand screening, RNA-based therapeutics delivered via nanobody platforms, and precision medicine approaches for stroke. This figure was created with BioRender (agreement number: JB293PX58P).

In parallel, single-cell and spatial transcriptomic analyses of human stroke tissue provide unprecedented resolution of GPCR expression dynamics across disease stages and cell types [148]. Together, these approaches offer critical guidance for identifying phase-specific and cell-selective GPCR targets with improved translational potential (Figure 4).

### 6.6. Perspectives: Toward Precision GPCR Targeting in Cerebrovascular Diseases

Collectively, these challenges highlight the need for a paradigm shift in GPCR-based therapeutic strategies for cerebrovascular diseases, from receptor-centric modulation toward phase-specific and cell-type-resolved targeting within the NVU. Advances in GPCR pharmacology and drug design are beginning to enable this transition.

Structural biology approaches, including cryo-electron microscopy and X-ray crystallography, have provided high-resolution insights into GPCR–ligand interactions, facilitating the rational development of subtype-selective and signaling-biased ligands that preferentially engage protective pathways while minimizing deleterious effects [149,150]. In parallel, emerging therapeutic modalities, such as nanobody-based biologics and RNA-based interventions, offer enhanced specificity and improved delivery to defined GPCR targets in the brain [151,152].

Precision targeting may be further supported by biomarker-guided strategies. Circulating GPCR ligands or cell-specific receptor expression patterns, such as A2AR levels on peripheral blood mononuclear cells, have shown promise as indicators of disease state and therapeutic responsiveness [153,154]. In addition, AI-assisted ligand screening and computational modeling are accelerating the discovery and optimization of selective GPCR modulators with improved pharmacokinetic and safety profiles [155].

Integrating these advances with human-relevant disease models and temporal knowledge of GPCR signaling may enable selective engagement of beneficial GPCR pathways while avoiding adverse vascular and inflammatory consequences. Such precision-oriented strategies are likely essential for translating GPCR biology into effective therapies for cerebrovascular diseases.

## Figures and Tables

**Table 1:** GPCR signaling mechanisms and translational relevance in cerebrovascular diseases.

GPCR	Major NVU Cell Types	Endogenous Ligand/ Representative Modulator	Ligand Type	Dominant Signaling Pathway	Phase- Dependent Effects	BBB Permeability	Clinical Development Status	Known Signaling Bias	References
**EP2/EP4**	VSMCs, neurons	PGE_2_	Agonist (endogenous)	Gs-cAMP-PKA	Acute: neurovascular coupling, ↑CBF; Dysfunction: neurovascular uncoupling	Indirect	Preclinical	Gs-biased	[48,49,50]
**P2Y12**	Microglia	ATP/ADP	Agonist (endogenous)	Gi-cAMP	Acute: microglial process extension, injury surveillance	N/A	Approved (antiplatelet, peripheral)	Gi-biased	[94,95,96]
**GPR4**	Endothelial cells	Protons (low pH)	Endogenous activator	G12/13-RhoA/Gs-cAMP	Acute: leukocyte adhesion, edema	N/A	Preclinical	G12/13-biased	[45,56,57]
**ETAR**	VSMCs, pericytes	ET-1/Bosentan	Agonist/antagonist	Gq-Ca^2+^	Acute: vasospasm, no-reflow; blockade improves CBF but may worsen viral injury	Limited	Approved (PAH)	Gq-biased	[51,52,53]
**CX3CR1**	Microglia	CX3CL1 (fractalkine)	Agonist (endogenous)	Gi-cAMP	Recovery: inflammation resolution, synaptic stabilization	N/A	Preclinical (stroke)	Context-dependent	[70]
**S1P1**	Endothelial cells, astrocytes	S1P/Fingolimod	Functional agonist	Gi-Rac1	Recovery: BBB repair, anti-inflammation	Yes	Approved (MS)	Functional agonism	[37,38,68,69]
**S1P4**	Endothelial cells	S1P	Agonist	G12/13-RhoA/Gi	Barrier-protective signaling	Unknown	Preclinical	Poorly defined	[59,97]
**A2AR**	Neurons, endothelial cells, microglia, pericytes	Adenosine/CGS21680/Istradefylline	Agonist/Antagonist	Gs-cAMP-PKA	Acute: ↑CBF, neuroprotection; Chronic: ↑BBB permeability, inflammation	Yes	Approved (PD); repurposing explored for stroke	Context-dependent	[60,61,62,63,64,65]
**β2AR**	VSMCs, neurons, astrocytes	Norepinephrine/Salbutamol	Agonist	Gs-cAMP; β-arrestin	Acute: vasodilation; Chronic: β-arrestin-mediated inflammation	Yes	Approved (non-stroke)	G protein/β-arrestin-biased	[66,67]
**α1AR**	VSMCs	Norepinephrine	Agonist	Gq-Ca^2+^	Acute: vasoconstriction, hypoperfusion	Limited	Approved (CV indications)	Gq-biased	[98,99]
**AT1R**	VSMCs, endothelial cells	Angiotensin II/Losartan	Agonist/Antagonist	Gq-Ca^2+^	Acute: vasoconstriction, hypoperfusion	Limited	Approved (hypertension)	Gq-biased	[54,55]
**GPR75**	VSMCs	20-HETE	Endogenous agonist	Gq-Ca^2+^-PKC	Acute: vasoconstriction, hypertension-related hypoperfusion	Unknown	Preclinical	Gq-biased	[100]
**GPER**	Endothelial cells	Estrogen/Aldosterone	Agonist	Gs-cAMP	Acute/Recovery: NO-mediated vasodilation	Indirect	Preclinical	Gs-biased	[101,102]
**GPR5C**	VSMCs	Orphan	Modulator	AT1R-Ca^2+^ facilitation	Acute: enhances Ang II-induced vasoconstriction	N/A	Preclinical	Context-dependent	[103]
**GPR5B**	Endothelial cells, VSMCs	Orphan	Modulator	Inflammatory GPCR networks	Chronic: vascular inflammation, metabolic stress	N/A	Preclinical	Poorly defined	[104]
**CXCR4**	Microglia, neurons, endothelial cells	CXCL12	Agonist	Gi-cAMP	Acute: inflammation, leukocyte recruitment; Recovery: repair signaling	Limited	Approved (oncology); preclinical stroke	Gi-biased	[105,106,107]
**PKR1/2**	Neurons	PK2	Agonist	Gs-cAMP/Gi-cAMP/Gq-Ca^2+^	Acute: pro-inflammatory; Context-dependent neuro-glial signaling	Unknown	Preclinical	Context-dependent	[78]
**CB2**	Microglia, immune cells	Endocannabinoids	Agonist	Gi-cAMP	Anti-inflammatory, neuroprotection	Yes	Preclinical	Gi-biased	[108]
**S1P2**	Endothelial cells	S1P	Agonist	G12/13-RhoA	Acute: BBB disruption	Unknown	Preclinical	G12/13-biased	[109,110]
**S1P3**	Endothelial cells	S1P	Agonist	G12/13-RhoA/Gq- Ca^2+^/Gi-cAMP	BBB protection in ICH via CCL2 suppression	Unknown	Preclinical	Context-dependent	[58,59]
**H1R/H2R**	Endothelial cells	Histamine	Agonist	Gq-Ca^2+^ (H1R)/Gs-cAMP (H2R)	Acute: ↑BBB permeability, capillary leakage	NA	Approved (allergy)	Receptor-specific	[111,112,113]
**H3R/H4R**	Endothelial cells, immune cells	Histamine	Agonist	Gi-cAMP	Acute/Chronic: immune cell trafficking, BBB modulation	Limited	Approved (non-CNS/immune)	Gi-biased	[111,113,114]
**TDAG8**	Immune cells	Protons (low pH)	Endogenous activator	Gs-cAMP-PKA	Acute: anti-apoptotic, neuroprotection	N/A	Preclinical	Gs-biased	[86,115]
**GPR68**	Neurons	Protons (low pH)	Endogenous activator	Gq-Ca^2+^-PKC	Acute: neuroprotection under acidosis	Yes	Preclinical	Gq-biased	[87]
**GPR37**	Neurons, astrocytes	Prosaposin	Agonist (endogenous)	Gi-cAMP	Recovery: neuronal survival, anti-inflammation	Yes	Preclinical	Gi-biased	[85,116]
**PAC1**	Neurons	PACAP	Agonist	Gs-cAMP-PKA/Gq-Ca^2+^-PKC	Recovery: neuroprotection, plasticity	Yes	Preclinical	Context-dependent	[71]
**B2R**	Neurons, endothelial cells	Bradykinin	Agonist	Gq-Ca^2+^-NO	Acute/Recovery: neuroprotection, angiogenesis	Limited	Approved (non-CNS)	Gq-biased	[83,117]
**A1AR**	Neurons, astrocytes	Adenosine	Agonist	Gi-cAMP	Acute: anti-excitotoxic; Excessive activation may impair perfusion	Yes	Preclinical (stroke)	Gi-biased	[118]
**CCR5**	Neurons, microglia	CCL5/Maraviroc	Antagonist	Gi-cAMP	Recovery: neuroplasticity, motor recovery	Limited	Approved (HIV); repurposing explored	Gi-biased	[88,89]
**5-HT1/2**	VSMCs, neurons	Serotonin	Agonist/Antagonist	Gi-cAMP (5-HT1)/Gq-Ca^2+^ (5-HT2)	Acute: cerebrovascular tone regulation	Limited	Approved (migraine)	Receptor-specific	[60,119,120,121,122]

## Data Availability

No new data were created or analyzed in this study. Data sharing is not applicable to this article.
